# Persistent homology analysis of brain transcriptome data in autism

**DOI:** 10.1098/rsif.2019.0531

**Published:** 2019-09-25

**Authors:** Daniel Shnier, Mircea A. Voineagu, Irina Voineagu

**Affiliations:** 1 Department of Mathematics and Statistics, University of New South Wales, Kensington, Sydney, New South Wales 2052, Australia; 2 Department of Biotechnology and Biomolecular Sciences, University of New South Wales, Kensington, Sydney, New South Wales 2052, Australia

**Keywords:** transcriptome, gene expression, topology, persistent homology, autism

## Abstract

Persistent homology methods have found applications in the analysis of multiple types of biological data, particularly imaging data or data with a spatial and/or temporal component. However, few studies have assessed the use of persistent homology for the analysis of gene expression data. Here we apply persistent homology methods to investigate the global properties of gene expression in post-mortem brain tissue (cerebral cortex) of individuals with autism spectrum disorders (ASD) and matched controls. We observe a significant difference in the geometry of inter-sample relationships between autism and healthy controls as measured by the sum of the death times of zero-dimensional components and the Euler characteristic. This observation is replicated across two distinct datasets, and we interpret it as evidence for an increased heterogeneity of gene expression in autism. We also assessed the topology of gene-level point clouds and did not observe significant differences between ASD and control transcriptomes, suggesting that the overall transcriptome organization is similar in ASD and healthy cerebral cortex. Overall, our study provides a novel framework for persistent homology analyses of gene expression data for genetically complex disorders.

## 1. Introduction

Autism spectrum disorders (ASDs), and more broadly neurodevelopmental disorders, are clinically as well as genetically heterogeneous conditions [1]. ASDs manifest with a combination of social interaction impairment and repetitive behaviours, accompanied by language deficits [2]. The clinical picture varies widely, with individuals at one end of the spectrum being severely impaired and needing permanent care, while at the other end of the spectrum, ASD patients can be highly functional. The clinical heterogeneity of ASDs is mirrored by genetic heterogeneity. Although ASDs are highly heritable (with population-based heritability estimates around 50% [3]), the genetic variants that underlie this heritability have proven difficult to identify [4]. Recent estimates suggest that hundreds of common and rare variants contribute to disease risk, and the combination of genetic variants differs widely between ASD individuals [4].

To investigate whether genetic variants converge onto a common set of molecular pathways at the level of gene expression, we and others have carried out gene expression studies of post-mortem brain tissue from ASD individuals [5–7]. These studies have identified genes differentially expressed between ASD and controls, highlighting a downregulation of neuronal synaptic genes and an upregulation of immune and inflammatory genes. Co-expression network analyses have also been employed to identify groups of genes that covary across the ASD and control samples, thereby being able to identify more subtle gene expression differences between ASD and control brain [6,7]. However, it remains unknown whether there are global differences between the brain transcriptomes of ASD cases and controls.

Genome-wide expression data are characterized by complex interdependencies and nonlinearities that are often missed by standard statistical methods. Topology has emerged as a powerful tool to analyse and interpret high-dimensional data, due to its ability to study properties that are robust against choice of coordinates, choice of metric and more generally continuous deformations, motivating its use in the analysis of transcriptome data [8]. Persistent homology introduced by Edelsbrunner *et al*. [9] aims to characterize essential topological features of an object. Therefore, it is rather intuitive to apply to spatial or temporal data. Persistent homology has been applied successfully for unsupervised learning on imaging data [10], including brain scan data in neurodevelopmental disorders [11]. However, few studies have explored it as a method to analyse gene expression data. To our knowledge, the application of persistent homology to gene expression has been limited to the analysis of time-course series [12] and assessing the effect of copy number variants on gene expression in cancer [13]. The latter study assessed the component of gene expression explained by the gene's spatial coordinates (i.e. chromosomal location).

**Figure 1.**
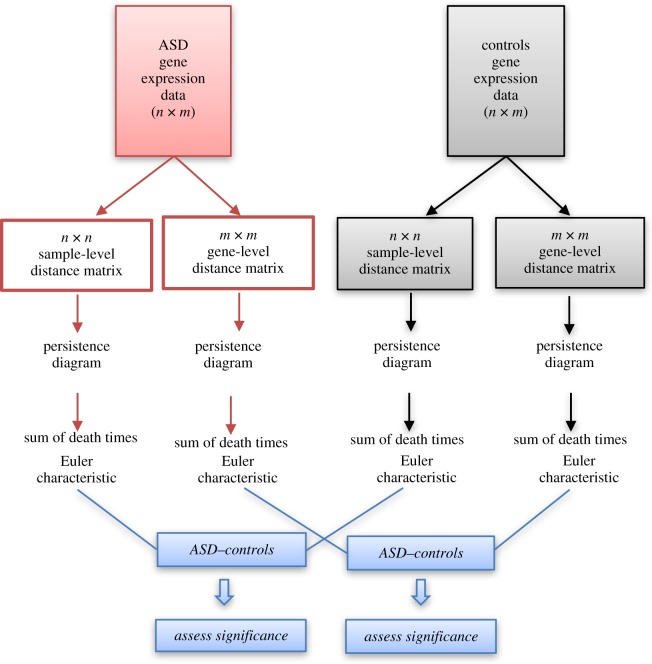
Study overview. For each gene expression dataset, the ASD and control groups were analysed by generating either a gene-level or a sample-level distance matrix (1-Pearson correlation). Distance matrices were used to compute persistence diagrams and their corresponding Betti number and Euler characteristic. The difference in these topological invariants between ASD and controls was then assessed for significance by random permutation of sample labels. (Online version in colour.)

Here we assess for the first time the application of persistent homology to gene expression data from individuals with a genetically complex disorder of unknown cause (i.e. without known chromosomal abnormalities). We apply this approach to two gene expression datasets from brain samples of individuals with idiopathic autism and matched controls [6,7]. The overall aim of our study (figure 1) was to better understand the global properties of gene expression data in the ASD and control groups. To this end, each gene expression dataset of *N* genes measured in *M* samples was conceptualized as either of the following.

(a) A cloud of *M* points in *N*-dimensional space, where each point designates a sample, and the distance between points represents inter-sample dissimilarity. Studying the topological properties of such a sample-level point cloud allows the identification of nonlinear relationships between samples. We compared the topological properties of the ASD and control sample point clouds in the two distinct gene expression datasets and found significant differences between ASD and controls, suggestive of increased heterogeneity among the ASD samples. This observation was replicated across both datasets.
(b) A cloud of *N* points in *M*-dimensional space, where each point designates a gene, and the distance between points represents the dissimilarity between genes. Persistent homology analysis of gene-level point clouds is conceptually a version of co-expression networks. Unlike standard co-expression networks, however, here we assessed the dissimilarity matrix using topological descriptors rather than hierarchical clustering. The use of topological descriptors such as the Euler characteristic (Material and methods) allowed us to globally assess the geometry of a gene expression dataset and to compare the topological properties between ASD and control transcriptomes. Interestingly, we did not observe a significant difference in the topological descriptors of ASD and control transcriptomes in either of the two datasets investigated, suggesting that the global transcriptome organization is not altered in ASD brain.

## 2. Material and methods

### 2.1. Overview of persistent homology

Classical homology investigates the topological properties of objects in a manner independent of scale, while persistent homology is a more recent topological data analysis method that examines changes in topological features in an object which evolves with respect to a scale parameter (reviewed in [14,15]). The objects we investigate using persistent homology are the point clouds formed by either samples or genes, and their associated pairwise distance matrices for each gene expression dataset.

#### 2.1.1. Simplices and simplicial complexes

In classical algebraic topology, objects are represented through simplicial complexes, which in turn are a collection of building blocks called simplices: a 0-simplex represents a point, a 1-simplex represents a segment, a 2-simplex represents a triangle, a 3-simplex represents a tetrahedron, etc. A simplicial complex consists of a finite set of simplices ‘glued’ together [16].

Vietoris–Rips simplicial complexes, which are used in the present study, are defined as follows. Given a set of points V ⊂ R^*n*^ (vertex set) and a distance metric *d* on R^*n*^, the Vietoris–Rips simplicial complex VR(V, *ε*) is defined as the simplicial complex where {*v*_0_, … ,*v_k_*} spans a *k*-simplex if *d*(*v_i_*, *v_j_*) ≤ *ε* for all 0 ≤ *i*, *j*, ≤ *k*.

#### 2.1.2. Persistent homology

In persistent homology, *ε* varies, which allows the assessment of topological invariants of an object at different scales. By choosing a sequence of epsilons that increase 0 < *ε*_0_ < *ε*_1_ < *ε*_2_ < … < *ε_n_* < … *ε*_max_ (where *ε*_max_ is the maximum distance between two points), we form an increasing sequence of simplicial complexes: VR(V, *ε*_0_) ⊂ VR(V, *ε*_1_) ⊂ VR(V, *ε*_2_) ⊂ … ⊂ VR(V, *ε_n_*) ⊂ … ⊂ VR(V, *ε*_max_). Considering the *k*th singular homology of these simplicial complexes, we form a sequence of maps between *H_k_* homology groups: *H_k_* (VR(V, *ε*_0_), *Z*) → *H_k_* (VR(V, *ε*_1_), *Z*) → *H_k_*(VR(V, *ε*_2_), *Z*) → … → *H_k_*(VR(V, *ε_n_*), *Z*) → … *H_k_*(VR(V, *ε*_max_), *Z*).

*Z* denotes integer numbers and here it represents the coefficients of the homology groups.

Roughly speaking, homology groups (*H_k_*) are composed of topological cycles; for example, *connected components* for *k* = 0 and *k*-dimensional *holes* for *k* > 0. The fact that homology groups have *Z* coefficients means that the operations applied to topological cycles are addition, subtraction and multiplication with integer numbers.

**Figure 2.**
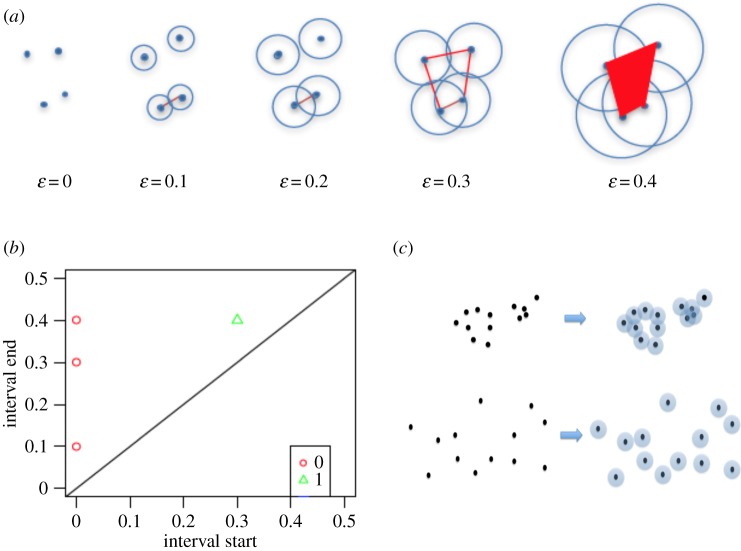
Schematic representation of basic persistent homology concepts. (*a*) Vietoris–Rips simplicial complexes VR(V, *ε*) formed by a cloud V of four points, at increasing *ε* values (*ε* is arbitrary, for illustration purposes). (*b*) Persistence diagram of the point cloud shown in (*a*). Zero-dimensional components are shown as red circles, one-dimensional components are shown as green triangles. For each component, the *x*-axis represents the *ε* value at which it is born (i.e. persistence interval start), and the *y*-axis represents the *ε* value at which it dies (i.e. persistence interval end). Persistent components are those located away from the diagonal. (*c*) Hypothetical examples of two point clouds of different degrees of heterogeneity. The number of points is the same in both point clouds, i.e. 13 points. The bottom example is more heterogeneous than the top example. Using circles of the same radius (*ε*/2), for the top example, we have an associated simplicial set with 2 connected components so the associated Vietoris–Rips complex VR(V, *ε*) has 2 connected components, while in the bottom example, we have an associated Vietoris–Rips complex VR(V, *ε*) with 13 connected components. Therefore, we have more connected components where the point cloud is more heterogeneous. (Online version in colour.)

The aim of persistent homology is to identify features of an object that are ‘persistent’ with respect to scale. We say that a topological cycle (i.e. a connected component or a hole of the space) is ‘born’ at *ε_n_* and ‘dies’ at *ε_m_* if the cycle belongs to *H_k_* (VR(V, *ε_n_*), *Z*), goes to zero in *H_k_* (VR(V, *ε_m_*), *Z*), its image is non-zero in all the intermediary homologies (i.e. the cycle exists from the moment it is born to the moment it dies) and the cycle does not belong to the image of the map *H_k_* (VR(V, *ε_n_*_−1_), *Z*) → *H_k_*(VR(V, *ε_n_*), *Z*). A simple example for a cloud of four points is shown in figure 2*a*.

In the case of connected components (i.e. for *k* = 0), all the connected components are born in the beginning at *ε* = 0. They die after several steps when two connected components merge forming a big connected component. In the end of the process, we are left with only one connected component that will survive to infinity. We can view Vietoris–Rips simplicial complexes indexed by *ε* as a collection of complexes {VR(V, *ε_k_*)}*_k_* with inclusion *x* : VR(V, *ε_i_*) → VR(V, *ε_i_*_+1_) between two consecutive steps.

The *k*-persistent homology with *Z*/2-coefficients of the persistent complex {VR(V, *ε_i_*)}_I_ is defined to be the set of Abelian groups {*H_k_* (VR(V, *ε_i_*), *Z*/2)}_i_ with inclusions *H_k_*(VR(V, *ε*_0_), *Z*/2) → *H_k_*(VR(V, *ε*_1_), *Z*/2) → … → *H_k_* (VR(V, *ε_n_*), *Z*/2) → … → *H_k_* (V R(V, *ε*_max_), *Z*/2).

*Z*/2 denotes the set of remainders to the division of integer numbers by 2, i.e. {0,1}. In the context of *H_k_* groups, the fact that their coefficients are limited to {0,1} means that the only operation applied to topological cycles is addition. The choice of *Z*/2 coefficients for *H_k_* groups is frequently used [15] as it gives a simplified version of persistent homology.

A way to represent the topological cycles that appear in persistent homology is through persistence diagrams (figure 2*b*), in which for each topological cycle *c*, the ‘birth’ *ε*_b_(*c*) is plotted on the *x*-axis and the ‘death’ *ε*_d_(*c*) value is plotted on the *y*-axis. Topological cycles of different dimensions are plotted in distinct colours. Persistent features are topological cycles located away from the diagonal.

Some useful summaries of a persistence diagram are the sum of the lengths of *k*-dimensional cycles (SL*_k_*) and the Euler characteristic. For a *k*-dimensional cycle *c* in a persistence diagram, we define the length of cycle *c* to be l(*c*) = *ε*_d_(*c*) − *ε*_b_(*c*) [17]. We denote the sum of the lengths *l*(*c*) of all *k*-dimensional cycles appearing in the persistence diagram by SL*_k_*, the sum of their birth times by SBT*_k_* and the sum of their death times by SDT*_k_*. Since all connected components (i.e. cycles of dimension 0) are born at *ε_b_* = 0, SL_0 equals_ SDT_0_.

Note that the topological invariant SDT_0_ can be interpreted as a measure of the heterogeneity of the initial vertex set V, which in our case is the point cloud associated with a dataset. Since a more heterogeneous group of points would have more connected components that survive longer, *a priori* it is expected that higher heterogeneity of a point cloud would result in larger SDT_0_ numbers. An exemplification of this concept is shown in figure 2*c*.

The *Euler characteristic* [17] of a persistent diagram in which we have cycles of dimension from 0 to *n* is defined as: *χ* = SL_0_ − SL_1_ + … +(−1)*^n^*SL*_n_*.

Because cycles of dimension more than 2 are very rare in our persistent homology diagrams, the Euler characteristic was computed using dimension 0, 1 and 2 cycles, i.e. *χ* = SL_0_ − SL_1_ + SL_2_. Thus, the Euler characteristic of a persistent diagram is an invariant that depends on zero cycles, but also on higher dimensional cycles.

### 2.2. Gene expression datasets

Gene expression data were obtained from two published studies: a microarray study which quantified the expression of 9934 genes, and an RNA-seq study which quantified the expression of 22 399 genes in cerebral cortex samples from ASD cases and controls [6,7]. Since the number of connected components can depend on the number of data points, we included the same number of ASD and control cerebral cortex samples from each dataset (29 per group for the microarray data and 82 per group for the RNA-seq data). This would allow us to compare the persistent homology groups between ASD and controls. For each dataset, ASD cases and healthy controls had been matched for age and gender in the original studies [6,7]. Further, there was no significant difference in age, post-mortem interval or RNA integrity numbers between autism and control cortex samples included in our analysis.

The microarray data have been quantile normalized and log2 transformed, and the RNA-seq data have been RPKM-normalized and log2 transformed.

### 2.3. Persistent homology analysis

For each gene expression dataset, we calculated (a) an inter-sample distance matrix and (b) an inter-gene distance matrix for ASD and control data separately, using 1 − *r* (*r*: Pearson correlation coefficient) as a dissimilarity measure. For sample point clouds, we constructed Vietoris–Rips complexes, based on the vertex set given by the points in each dataset. Persistent homology was computed using the *pHom* function in the *pHom* R package [18] (https://github.com/cran/phom/blob/master/man/pHom.Rd), and persistence diagrams were plotted using the *plotPersistenceDiagram.* For gene-point clouds, which included thousands of points, the Vietoris–Rips complexes are extremely large, and therefore we used an alternative implemented in the *pHom* function, the lazy-witness construction, with landmark_set_size = 20. The *pHom* function [18], similarly to most other persistent homology algorithms [16], considers persistent homology with *Z*/2-coefficients.

### 2.4. Mahalanobis distance-based analysis

Mahalanobis distance (MD) was calculated as previously described [19]:

$$\mathrm{MD}(x_{i},\;x_{c})=\sqrt{{\left(x_{i}-x_{c}\right)}^{T}\Psi^{-1}(x_{i}-x_{c})},$$

where *x_i_* is the vector of gene expression values in ASD sample *i*, *x*_c_ is the vector of gene expression means across controls and *ψ*^−1^ is the inverse of the covariance matrix estimated from control samples. Since some of the covariance matrices did not have an inverse, we calculated the Moore–Penrose generalized inverse as implemented in the *pinv* function in the *pracma* (Practical Numerical Math Functions) R package (https://cran.r-project.org/web/packages/pracma/).

Using all genes in the microarray data, the sum of squared MD (SSMD) was calculated for ASD samples and compared with values obtained by 1000 random permutations of group labels. False discovery rate (FDR) was defined as the ratio of random permutation values that were more extreme than the observed SSMD value. When attempting to carry out the same analysis using the larger RNA-seq dataset on a powerful computer (2 × 2.66 GHz 6-Core, 64GB RAM), the MD analysis of the RNA-seq data took over 13 h per computation, and thus a permutation-based analysis was not possible.

For the MD analysis of KEGG gene sets, SSMD was calculated as above for ASD samples, using the genes within each set, rather than the entire transcriptome. For each gene set, the observed SSMD value was compared with 100 randomly sampled gene sets of the same size.

All data analysis codes are available as a Github repository: https://github.com/Voineagulab/Persistent_Homology_ASD_Brain/.

## 3. Results

We investigated the topological properties of gene expression data in the cerebral cortex in autism using two published datasets, generated with two distinct methods: microarays [6] and RNA-sequencing [7].

There is generally good agreement between gene expression measurements by microarrays and RNA-seq [7], with RNA-seq data having the additional advantages of being more sensitive and having a wider dynamic range. A highly significant overlap has been observed between genes identified as differentially expressed in the two studies [7]. Analysing two datasets generated with different methods allowed us to assess if our observations are replicable and independent of the technical properties of each method.

**Figure 3.**
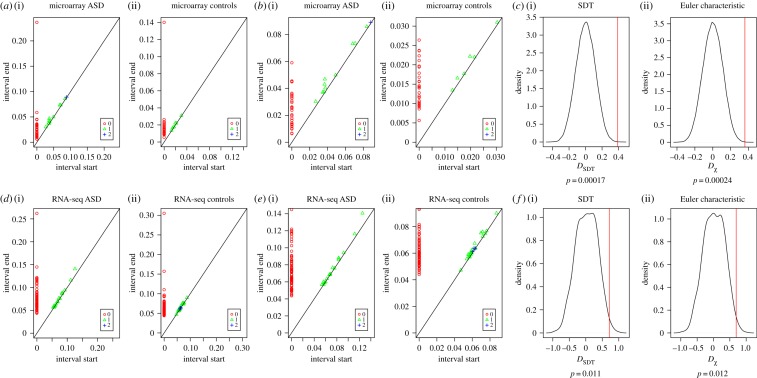
Persistent homology analysis of sample-level point clouds. (*a*) Persistence diagrams of ASD and control groups, based on the microarray dataset. (*b*) The same persistence diagrams as in (*a*) are plotted with a zoomed-in *y*-axis, to better visualize components with dimension greater than 0. (*c*) (i) Density plot of SDT difference between ASD and controls (*D*_SDT_) generated by 100 000 random permutations of sample labels. Vertical red line: observed *D*_SDT_ value. (ii) Density plot of Euler characteristic difference between ASD and controls (*D_χ_*) generated by 100 000 random permutations of sample labels. Vertical red line: observed *D_χ_* value. (*d*) Persistence diagrams of ASD and control groups, based on the RNA-seq dataset. (*e*) The same persistence diagrams as in (*d*) are plotted with a zoomed-in *y*-axis, to better visualize components with dimension greater than 0. (*f*) (i) Density plot of SDT difference between ASD and controls (*D*_SDT_) generated by 1000 random permutations of sample labels. Vertical red line: observed *D*_SDT_ value. (ii) Density plot of Euler characteristic difference between ASD and controls (*D_χ_*) generated by 1000 random permutations of sample labels. Vertical red line: observed *D_χ_* value. (Online version in colour.)

To assess nonlinear relationships between sample (dis)similarity among ASD and control cerebral cortex samples, we constructed persistent homologies based on Vietoris–Rips simplicial complexes (Material and methods) for ASD and control data separately. The sample-level point clouds consisted of 29 ASD and 29 control samples for the microarray data and of 82 ASD and 82 control samples for the RNA-seq data. We observed that the most persistent features of these point clouds were connected components (zero-dimensional; figure 3*a,b*). This observation held true regardless of whether the data were from ASD or controls, or whether they were generated by microarrays or RNA-seq (figure 3*d,e*). Higher-dimension topological cycles were also identified, with the maximum dimension being 2 (figure 3). However, all of the one-dimensional and two-dimensional topological cycles were transient, i.e. they were ‘born’ and ‘died’ at very close *ε* values.

Since connected components were persistent features, we next compared their properties between ASD and control data. To summarize all the connected components within a dataset, we computed two topological invariants: the sum of the death times of connected components (i.e. SDT_0_; Material and methods) and the Euler characteristic (*χ*; Material and methods), which also takes into account one-dimensional and two-dimensional topological cycles. We found that STD_0_ASD_ was higher than SDT_0_Control_, and *χ*__ASD_ was higher than *χ*__Control_, an observation replicated across the microarray and RNA-seq datasets.

To test the statistical significance of this observation, we carried out random permutations of sample labels (figure 3). We recomputed the persistent homology, as well as SDT_0_ and *χ* at each permutation. We then calculated *D*_SDT_ = SDT_0_ASD_ − SDT_0_Control_, and *D_χ_* = *χ*__ASD_ − *χ*__Control_ at each permutation. The FDR was calculated as the proportion of random permutation trials giving a more extreme *D*_SDT_ or *D_χ_* value than the observed values. For both microarray and RNA-seq data, the differences between ASD and controls were highly significant, using either of the two topological invariants (figure 3*c*,*f*). We should mention that in this particular case, where most one-dimensional and two-dimensional components are very transient, the Euler characteristic value is very close to the SDT_0_.

What is the interpretation of a higher sum of the death time number observed for ASD data compared to control gene expression data? We propose that the higher sum of the death time number reflects higher heterogeneity within the ASD group (Material and methods). Remarkably, a similar observation has been made on FDG-PET brain imaging data from 26 ASD and 11 control individuals, where SDT_0_ was higher for ASD data compared to controls [11].

We also assessed whether the increased heterogeneity of gene expression data in ASD samples was contributed by specific subsets of genes or whether it was a property of the entire transcriptome. To this end, we used functional gene sets curated in the KEGG database (Kyoto Encyclopedia of Genes and Genomes [20]). Of the 186 gene sets included in the KEGG database, 104 had at least half of the genes expressed in our microarray brain data. For each of the 104 gene sets, we assessed whether *D*_SDT_ or *D_χ_* was significantly different from 100 randomly selected sets of genes of the same size. This analysis identified one gene set as significant: the MAPK signalling pathway (FDR less than 0.01). The result was replicated in the RNA-seq dataset (FDR less than 0.01).

We next investigated the topological properties of expression (dis)similarities between genes, within the ASD and control groups, using the same microarray and RNA-seq datasets. Here we assessed the topological invariants of point clouds consisting of 9934 genes for the microarray dataset and 22 399 genes for the RNA-seq data. Similarly to the sample-level analysis, the ASD and control data were assessed separately. Owing to the large number of points in the gene-level point clouds, we used an approximation of Vietoris–Rips complexes (Material and methods) and assessed statistical significance using 1000 random permutations of sample labels.

The gene-level point clouds showed somewhat more complex topological features than the sample-level point clouds: in addition to persistent zero-dimensional connected components, we also observed a few persistent one-, two- and three-dimensional components (electronic supplementary material, figure S1). However, we did not observe any significant difference in the sum of the death times or Euler characteristic between ASD and controls, suggesting that globally, there are no significant differences in transcriptome organization between ASD and controls (electronic supplementary material, figure S1). This result is consistent with our earlier observations based on co-expression network analyses [6].

To compare our results with previously reported methods of assessing the dispersion of gene expression data in ASD brain, we employed an MD-based approach [19]. MD has previously been used to assess the distance between a vector of gene expression values from an ASD sample, and the mean of control samples [19]. One can then ask whether a summary MD value of all ASD samples (such as SSMD) is either (a) significantly higher than expected by chance, through random permutations of group labels or (b) is significantly higher within a set of genes compared to randomly sampled sets of genes of the same size. We found that SSMD of ASD samples was significantly higher than expected by chance (FDR less than 0.001, 1000 random permutations of group labels; Material and methods) using the microarray dataset, confirming the persistent homology-based result. Owing to the larger gene expression matrix size and the need to calculate the inverse of the covariance matrix, MD analysis was not computationally feasible for the RNA-seq dataset (13 h per computation; Material and methods). For the same reason, the MD-based transcriptome-wide gene-level analysis could not be carried out.

We also applied the MD-based approach to functional gene sets from the KEGG database. Using the microarray data, we identified 3 gene sets showing significantly higher SSMD for ASD samples than 100 randomly sampled sets of genes of the same size: ‘MAPK signalling pathway’, ‘pathways in cancer’ and ‘cell cycle’ (FDR less than 0.01). The result for the first two gene sets, but not for ‘cell cycle’, was replicated in the RNA-seq dataset.

Overall, we found that the differences between ASD and control gene expression data detected by persistent homology analysis were confirmed by the MD-based analysis, but persistent homology handled better the large datasets.

## 4. Discussion

Here we applied persistent homology methods to investigate the global properties of gene expression data from autistic individuals and matched controls in two distinct datasets (a microarray and an RNA-seq dataset). Unlike previous persistent homology studies of gene expression data [13], our purpose was not to classify samples based on their gene expression profiles, but rather to investigate the properties of gene expression data within each phenotypic group.

By assessing topological invariants of the inter-sample distance matrices, we found that both the SDT_0_ and the Euler characteristic were significantly higher for the ASD group, in both datasets studied. This observation demonstrates that gene expression data from ASD individuals are more heterogeneous than gene expression from controls, based on inter-sample relationships. Quantifying heterogeneity is an important question for autism genetics. In addition to its clinical heterogeneity, ASD is also heterogeneous at the level of DNA sequence variation, with most ASD patients carrying a unique combination of DNA sequence changes [17]. Our results indicate that heterogeneity, a hallmark clinical property of ASD, is reflected at the molecular level of gene expression. Gene expression heterogeneity in ASD brain samples suggests potential dysfunction across multiple transcriptional regulatory proteins, with the specific proteins impacted being different in distinct individuals. This notion is consistent with the fact that variants in genes encoding more than 12 transcription regulatory proteins have been associated with ASD [21].

The MAPK signalling pathway has been previously implicated in ASD [22]. Patients with mutations in genes encoding members of the MAPK pathway have increased incidence of ASD [23]. Our data suggest that MAPK signalling pathway may also be impacted in patients with idiopathic ASD at a transcriptional level.

Furthermore, our study provides a framework based on persistent homology that allows the quantification of heterogeneity of high-dimensional data that can be further applied for comparisons of multiple types of genomic data (such as gene expression, DNA methylation and sequence variants). Such comparisons could address an outstanding question: which molecular layers contribute to the clinical heterogeneity of ASD and will be the focus of our future work.

We also assessed the gene pairwise distance matrices using persistent homology, which can be thought of as a topology-based co-expression network approach. Since we are computing topological invariants, we used 1-Pearson correlation coefficient as the distance matrix, rather than the topological overlap measure which is implemented in weighted gene co-expression network analyses [24,25]. Co-expression networks aim to group genes into sets of co-expressed genes (i.e. co-expression modules) but do not commonly compute descriptors that allow a global characterization of the topology of the network. Using either the SDT_0_ or the Euler characteristic of the gene-level point clouds, we did not observe any significant difference between ASD and control groups, showing that the ASD and control networks have similar topological properties. This result is consistent with our initial observations based on co-expression networks, where we observed significant overlap in the modules detected in the ASD and control networks [6]. A recent paper took a related approach, using the bottleneck distance between persistence diagrams, to assess (dis)similarities between co-expression networks from *Arabidopsis* after exposure to multiple types of stressors [26].

Persistent homology analyses of gene expression data are still in their infancy, and to our knowledge, our study is the first to apply persistent homology for brain co-expression networks. Further methodological developments are required in order to harness the full power of topological analyses for co-expression networks. In particular, the development of methods for assigning genes to higher dimensional components would facilitate extracting co-expression modules from topology-based networks.

## Supplementary Material

Supplementary Figure

## Data accessibility

Input data and data analysis code are available as a Github repository: https://github.com/Voineagulab/Persistent_Homology_ASD_Brain/.
