# Supplementary figures and images for: Persistent homology analysis of brain transcriptome data in autism
Source: J R Soc Interface. 2019 Sep 25;16(158):20190531. doi: 10.1098/rsif.2019.0531 (PMC6769309; doi:10.1098/rsif.2019.0531)

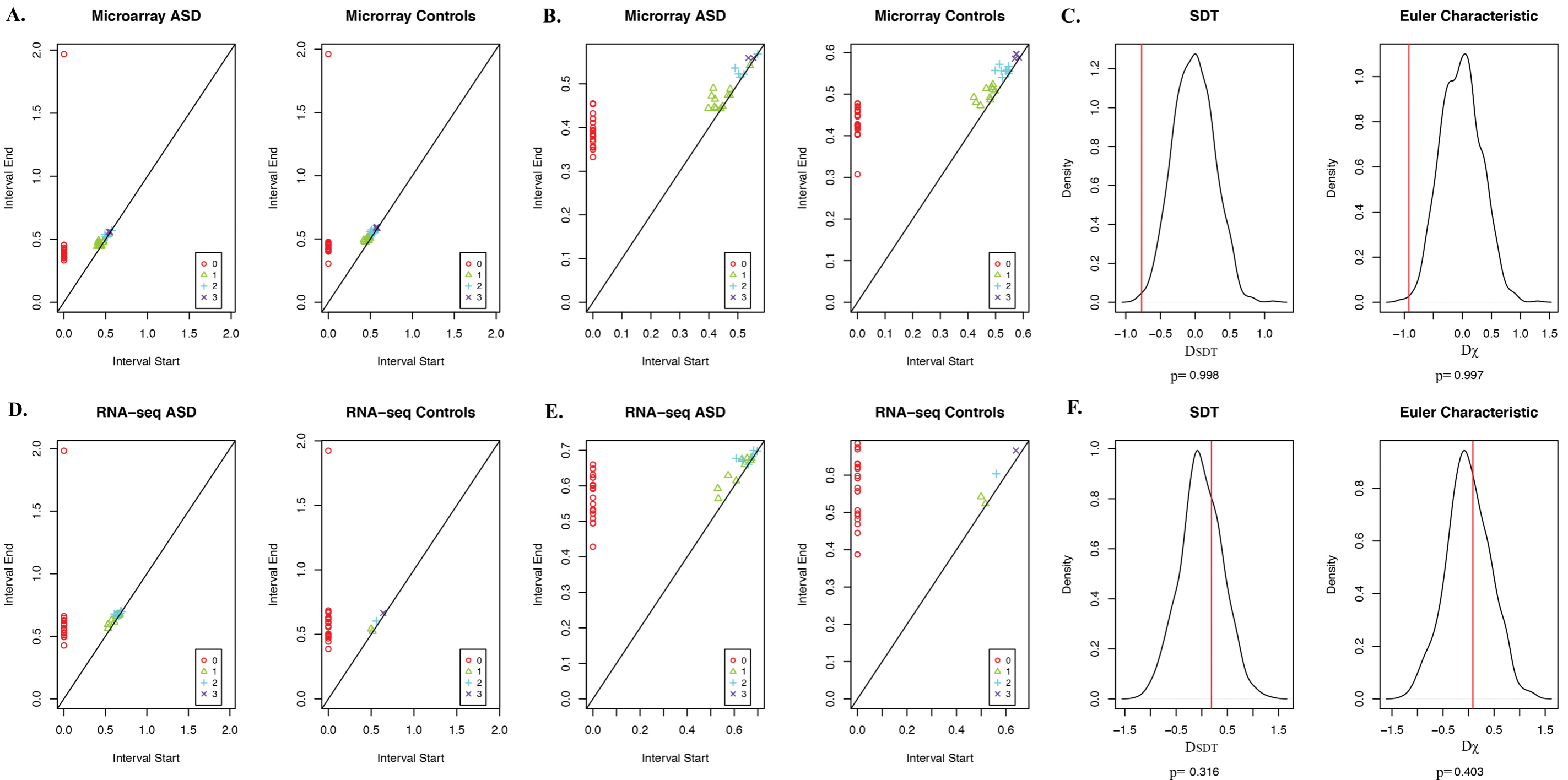

Supplement: Supplementary Figure [file rsif20190531supp1.pdf]
